# A-Type GABA Receptor as a Central Target of TRPM8 Agonist Menthol

**DOI:** 10.1371/journal.pone.0003386

**Published:** 2008-10-13

**Authors:** Xiao-Bing Zhang, Peng Jiang, Neng Gong, Xiao-Ling Hu, Da Fei, Zhi-Qi Xiong, Lin Xu, Tian-Le Xu

**Affiliations:** 1 Institute of Neuroscience and State Key Laboratory of Neuroscience, Shanghai Institutes for Biological Sciences, Chinese Academy of Sciences, Shanghai, China; 2 Laboratory of Learning and Memory, Kunming Institute of Zoology, Chinese Academy of Sciences, Kunming, China; University of Granada, Spain

## Abstract

Menthol is a widely-used cooling and flavoring agent derived from mint leaves. In the peripheral nervous system, menthol regulates sensory transduction by activating TRPM8 channels residing specifically in primary sensory neurons. Although behavioral studies have implicated menthol actions in the brain, no direct central target of menthol has been identified. Here we show that menthol reduces the excitation of rat hippocampal neurons in culture and suppresses the epileptic activity induced by pentylenetetrazole injection and electrical kindling *in vivo*. We found menthol not only enhanced the currents induced by low concentrations of GABA but also directly activated GABA_A_ receptor (GABA_A_R) in hippocampal neurons in culture. Furthermore, in the CA1 region of rat hippocampal slices, menthol enhanced tonic GABAergic inhibition although phasic GABAergic inhibition was unaffected. Finally, the structure-effect relationship of menthol indicated that hydroxyl plays a critical role in menthol enhancement of tonic GABA_A_R. Our results thus reveal a novel cellular mechanism that may underlie the ambivalent perception and psychophysical effects of menthol and underscore the importance of tonic inhibition by GABA_A_Rs in regulating neuronal activity.

## Introduction

Gamma-aminobutyric acid (GABA) is the major inhibitory neurotransmitter in the mammalian brain. Its principal action is to activate ionotropic A-type GABA receptors (GABA_A_Rs), leading to an inward flow of Cl^−^ and a hyperpolarizing postsynaptic response. The GABAergic transmission shapes neural activity via two spatially and temporally unique modes of inhibition [1]. The phasic (or synaptic) inhibition results from high-level GABA transients associated with evoked release of GABA, which activates synaptic GABA_A_Rs, whereas the tonic inhibition is caused primarily by ambient extracellular GABA acting on extrasynaptic high-affinity GABA_A_Rs [2], [3]. Previous studies in brain slices [2], [4] and neuronal cultures [5], [6], and *in vivo* [7] have shown that different GABA_A_R subtypes are responsible for mediating tonic inhibition, depending on brain regions and cell types [1], [8]. Recent studies suggest that tonic inhibition may regulate neural network excitability [2] and information processing [7]. Impairment of tonic inhibition may also contribute to pathological states such as chronic epilepsy [9]. Therefore, the enhancement of GABAergic tonic inhibition is a promising therapeutic approach for diseases involving network hyper-excitability.

(−)-Menthol is the best-known monoterpene extracted from the essential oil of the genus *Mentha* of the Lamiaceae family. Because of its pleasant flavor and aroma, and its cooling-anesthetic effect, menthol is used in many confectionary goods, pharmaceuticals, oral health care products, cosmetics, tea and tobacco products [10]. Menthol is also a primary activator of the cold- and menthol-sensitive TRPM8 channels [11], [12]. It facilitates glutamate release from sensory neurons by increasing intracellular Ca^2+^ level via activation of TRPM8 [13], [14], leading to modulation of peripheral nociception [15], [16]. Although behavioral studies have implicated menthol actions in the central nervous system (CNS) [17], no direct central target of menthol has been identified. In this study, we demonstrated the central actions of menthol on hippocampal neurons and showed a specific function of menthol in suppressing the excitation of hippocampal neurons by enhancing tonic GABA inhibition.

## Results

### Menthol suppresses neuronal excitation in hippocampal cultures

**Figure 1:**
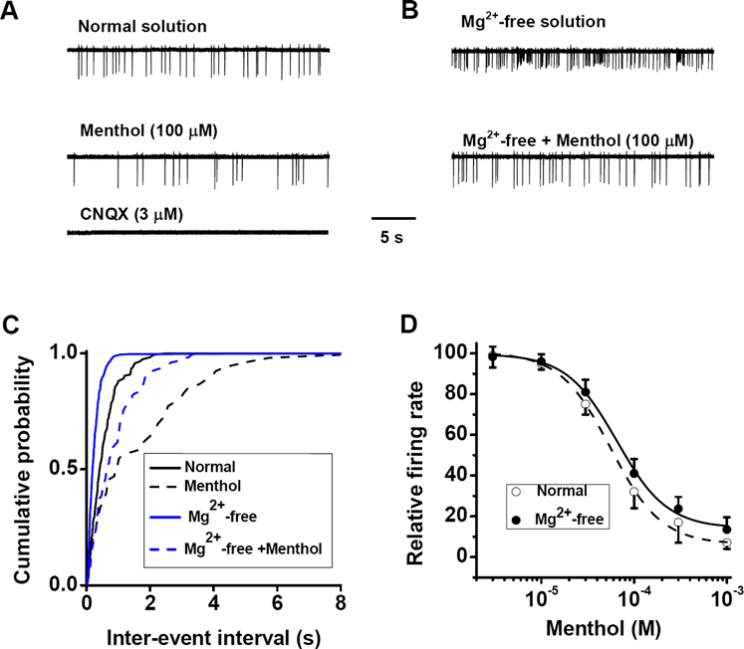
The inhibitory effect of menthol on synaptically driven spiking in hippocampal cultures. (A) Typical traces of spontaneous spiking from one cell in the presence or absence of menthol. (B) Typical traces of zero Mg^2+^-induced spiking from the same cell in the presence or absence of menthol. (C) The distribution of interspike interval obtained from *A* and *B* showing the effect of menthol on spontaneous spiking and elevated spiking. (D) Concentration-dependent inhibition of menthol on spontaneous and zero Mg^2+^-induced firing rate. (*n* = 6).

To explore the effect of menthol in central neurons, we first examined the menthol's effect on neuronal firing properties, using cell-attached voltage-clamp recording [18], [19] from cultured hippocampal neurons. Cultured neurons 12–16 day *in vitro* (DIV) had established functional synaptic connections and exhibited spontaneous spiking in the standard recording solution, with a mean firing rate of 1.6±0.4 Hz. This spontaneous spiking is synaptically driven, since it was completely abolished by bath addition of 6-cyano-7-nitroquinoxaline-2,3-dione (CNQX, 3 µM), a specific antagonist of alpha-amino-3-hydroxy-5-methyl-4-isoxazolepropionic acid (AMPA) subtype of glutamate receptors (Fig. 1A). Application of menthol dose-dependently reduced the firing rate, with an IC_50_ of about 54±5 µM (Fig. 1D). The menthol-induced reduction of spiking frequency was also shown by the rightward shift in the distribution of interspike intervals (Fig. 1C). Reducing the extracellular concentration of Mg^2+^ induces hyper-excitation in both hippocampal slices [20] and cell cultures [21] by enhancing glutamatergic transmission through elevated evoked glutamate release and increased activity of *N*-methyl-D-aspartate (NMDA) subtype of glutamate receptors. We found that the firing rate was significantly increased following the perfusion with the Mg^2+^-free solution (Fig. 1B and 1C), and menthol application dose-dependently reduced the firing rate with an IC_50_ (64±6 µM) similar to that found above for the suppression effect in standard recording solution (Fig. 1D). Thus menthol suppresses both spontaneous spiking and hyperactivity in hippocampal cultures.

### Synergistic activation of GABA_A_Rs by menthol and GABA

**Figure 2:**
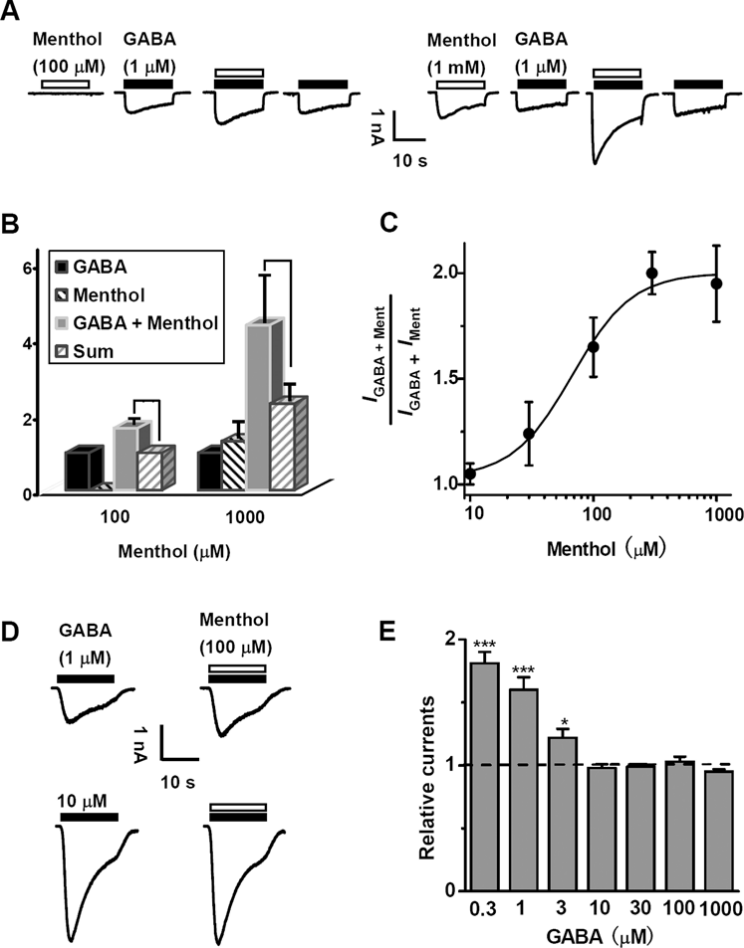
Synergistic activation of GABA_A_Rs by menthol and GABA. (A) Typical traces showing the currents evoked by 1 µM GABA, 100 µM menthol, 1 mM menthol, 1 µM GABA plus 100 µM menthol and 1 µM GABA plus 1 mM menthol. (B) Histograms showing relative *I*_GABA_, *I*_ment_, *I*_GABA+ment_. (C) The concentration-response relationship of synergic action of menthol with 1 µM GABA. (D) Typical traces showing the current evoked by different concentrations of GABA in the absence or presence of 100 µM menthol. (E) Statistic data showing the relative currents induced by 100 µM menthol plus various concentrations of GABA. * P<0.05 and *** P<0.001, compared with *I*_GABA_ in the absence of menthol.

**Figure 3:**
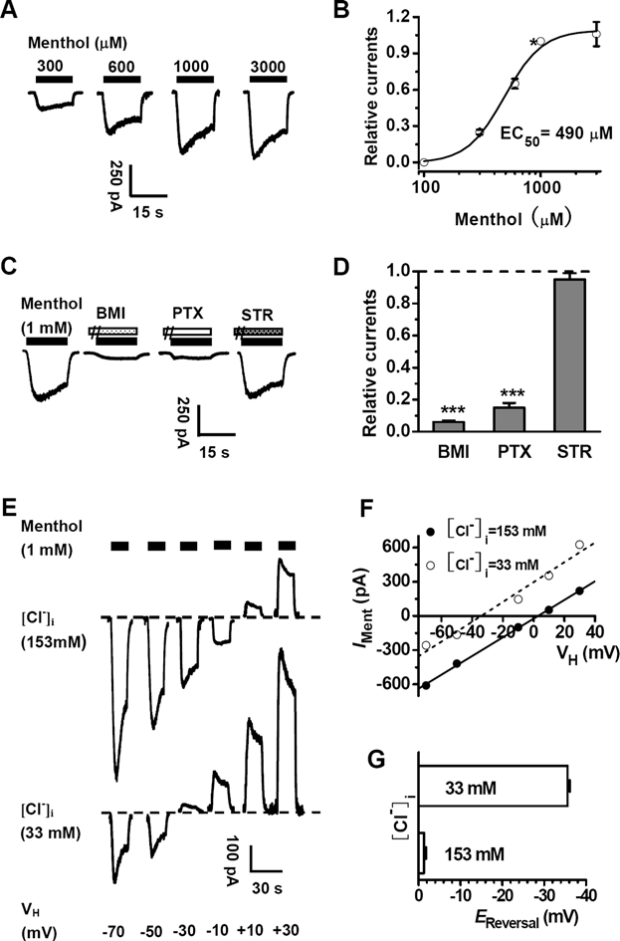
Pharmacological and electrophysiological properties of menthol-activated currents (*I*_ment_) in cultured hippocampal neurons. (A) Typical traces showing the currents induced by various concentration of menthol. (B) Concentration-response relationship of *I*_ment_ in cultured hippocampal neurons. All peak currents are normalized to the peak amplitude evoked by 1 mM menthol (*). Symbols represent the average response for 6–13 neurons. (C) Typical traces of *I*_ment_ evoked by 1 mM menthol in the absence or presence of 10 µM BMI (*n* = 15), 100 µM PTX (*n* = 5) and 1 µM STR (*n* = 15). (D) Summary results from all experiments similar to that shown in *C* showing the relative *I*_ment_ in the presence of BMI, PTX and STR. Dashed line indicates the control values without antagonist treatment. (E) Typical traces showing *I*_ment_ evoked by 1 mM menthol at various holding potentials (V_H_) with [Cl^−^]_i_ of 153 mM (upper traces) and 33 mM (lower traces). (F) The current-voltage relationships of *I*_ment_ in the condition of [Cl^−^]_i_ of 153 mM and 33 mM, respectively. The reversal potential of *I*_ment_ moved toward hyperpolarizing direction by lowering [Cl^−^]_i_. (G) Summary results showing the reversal potentials for *I*_ment_ in 153 mM [Cl^−^]_i_ and 33 mM [Cl^−^]_i_, respectively.

The suppression of neuronal excitation by menthol was not due to its effect on intrinsic membrane excitability of neurons. In the presence of a cocktail of transmitter receptor antagonists, including CNQX (10 µM), D-AP5 (20 µM, for NMDA receptors), bicuculline (BMI, 10 µM, for GABA_A_Rs) and strychnine (STR, 1 µM, for glycine receptors), we found that the firing frequencies of cultured hippocampal neurons induced by the same set of step-depolarization currents were identical before and after addition of menthol (300 µM, Fig. S1). Although TRPM8 protein is present in sensory neurons [11], we detected no TRPM8 mRNA in either cultured hippocampal neurons or rat hippocampus tissue of rats (Fig. S2), consistent with the idea that menthol regulates neuronal function via a mechanism independent of TRPM8. Based on the finding that menthol potentiates the response of recombinant GABA_A_Rs expressed in *Xenopus* oocytes [22], we examined the effect of menthol on the activation of GABA_A_Rs in hippocampal neurons. Menthol by itself had no effect on the resting membrane current (Fig. 2A and 2B) at low concentrations (≤100 µM), but it dose-dependently induced inward currents (*I*_Ment_) at higher concentrations (EC_50_ = 490 µM, Fig. 3). However, at 100 µM, menthol produced a marked enhancement of GABA-evoked current when co-applied with GABA (1 µM, Fig. 2B). The enhancement was quantified by measuring the peak amplitude of the membrane current evoked by a 20-s pulse of GABA alone (*I*_GABA_) and after co-application of GABA and menthol of different concentrations (*I*_GABA+Ment_) in the same cell (Fig. 2B and 2C). The results indicate that the threshold concentration of menthol for enhancing *I*_GABA_ was between 10–30 µM. The finding that the magnitude of *I*_GABA+Ment_ at high menthol concentrations (0.3 and 1 mM) was larger than the arithmetic sum of *I*_GABA_ and *I*_Ment_ is also consistent with a menthol-induced enhancement of *I*_GABA_. Importantly, *I*_Ment_ was virtually abolished by BMI (10 µM) and picrotoxin (PTX, 100 µM), but not by STR (1 µM) (Fig. 3), similar to that found for *I*_GABA_ or *I*_GABA+Ment_ (data not shown). Furthermore, the reversal potential for *I*_GABA+Ment_ was not significantly different from that found for *I*_GABA_ (data not shown), and that of *I*_Ment_ depended on internal Cl^−^ concentration in the same manner as that of *I*_GABA_ (Fig. 3). Together, these results suggest that menthol reduce neuronal excitation by specifically enhancing GABA_A_R-mediated inhibition.

The ambient GABA in the cerebral spinal fluid is estimated to be 0.8–2.9 µM [23], a concentration that may produce tonic neuronal inhibition by activating slowly-desensitizing extrasynaptic high-affinity GABA_A_Rs [24]. However, the GABA concentration in the synaptic cleft can reach millimolar levels during GABAergic transmission [25]. When the effect of menthol on GABA-induced currents in cultured hippocampal neurons was measured over a wide range (1–1000 µM) of GABA concentrations, we found that the enhancing effect on the peak current amplitude occurred only when GABA concentration was ≤3 µM (Fig. 2D and 2E), suggesting that menthol preferentially acts on extrasynaptic high-affinity GABA_A_Rs.

### The enhancement of menthol on tonic GABA currents in CA1 neuron of rat hippocampus

**Figure 4:**
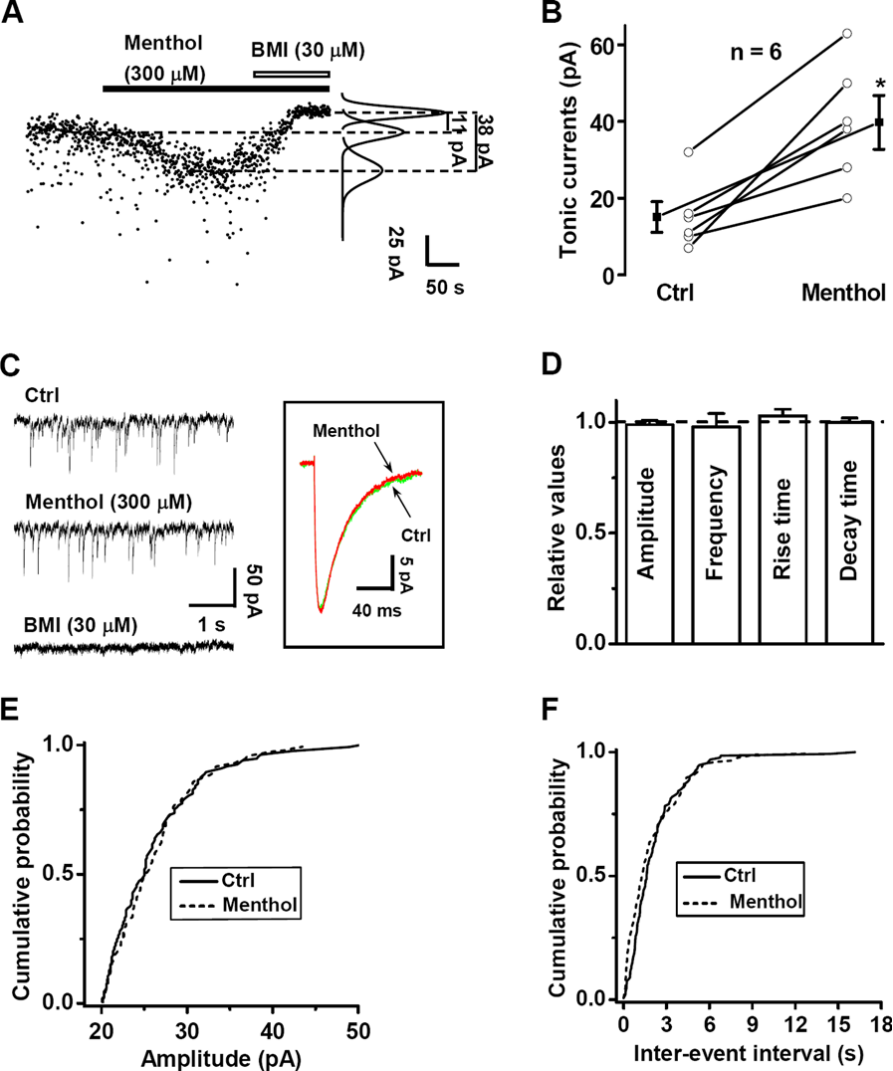
Selective enhancement of GABA_A_R-mediated tonic currents by menthol. (A) Voltage-clamp recordings from CA1 pyramidal neurons showing GABAergic tonic currents in the absence and presence of 300 µM menthol. The tonic current plotted at 500-ms intervals was revealed by 30 µM BMI. (B) Summary data showing the change of tonic currents by 300 µM menthol (*n* = 6, **P<0.01, compared with the control group without menthol treatment, paired *t*-test). (C) Representative traces showing GABAergic mIPSCs in the absence or presence of 300 µM menthol. Averaged mIPSCs in the absence or presence of 300 µM menthol are shown in the pane (D) Summary data showing normalized amplitude, frequency and kinetics of GABAergic mIPSCs in the presence of menthol (*n* = 14). Dashed line indicates the control values without menthol treatment. (E) Normalized cumulative curves showing the effect of menthol on the amplitude of GABAergic mIPSCs from the sample neuron. (F) Normalized cumulative curves showing the effect of menthol on the frequency of GABAergic mIPSCs from the sample neuron.

The above findings that menthol preferentially potentiated GABA_A_R-mediated currents at low GABA concentrations prompted us to examine the GABA_A_R-mediated tonic currents in hippocampal slices. Under the condition of high Cl^−^ concentration (147 mM) in the whole-cell recording pipette, the basal membrane current of CA1 pyramidal neurons underwent an upward shift following bath application of BMI, revealing the tonic GABA_A_R-mediated current (Fig. 4A and 4B). Application of menthol (300 µM) induced the downward shift of the membrane current, which was eliminated by subsequent application of BMI. This is consistent with notion that menthol had potentiated the action of endogenous tonic GABA. Interestingly, we found that menthol at the same concentration had no effect on the peak amplitude, rise time, decay time and frequency of spontaneous inhibitory postsynaptic currents (mIPSCs) (Fig. 4C–F). This is in line with the above finding in cultured hippocampal neurons that menthol selectively enhances tonic rather than synaptic (phasic) inhibition of GABA.

Further support of a specific action of menthol on GABAergic inhibition came from the analysis of spontaneous miniature excitatory post-synaptic currents (mEPSCs). In cultured hippocampal neurons, neither glutamate-activated macroscopic currents nor the amplitude and frequency of mEPSCs were affected by menthol (Fig. S3). These results further suggest that menthol reduced hippocampal neuronal excitation through a specific enhancement of GABAergic inhibition. Together with the finding that menthol mimics GABA by directly activating GABA_A_Rs at the high concentration range of 0.3 to 3 mM in cultured hippocampal neurons (Fig. 3), our data suggest that menthol could activate and sensitize GABA_A_Rs and thereby regulate hippocampal neuronal excitation.

### Inhibition of menthol on *in vivo* network hyper-excitability

**Figure 5:**
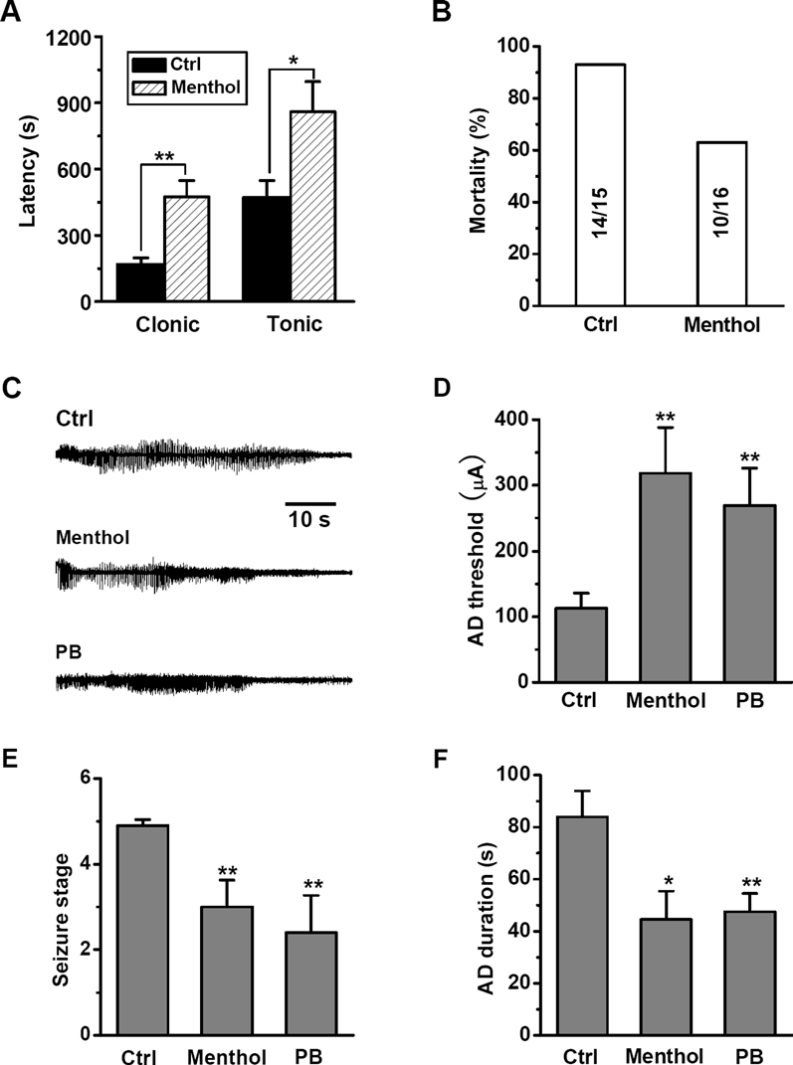
Effects of menthol on seizures of PTZ treated mice and hippocampal kindled rats. (A) Effect of menthol on the latency from PTZ injection to the clonic (**P<0.01 compared with Ctrl, One-Way ANOVA) and tonic (*P<0.05 compared with Ctrl) convulsion. (B) Effect of menthol on mortality in mice after PTZ injection. (C) Typical traces showing the effect of menthol and PB on the afterdischarge in fully kindled rats. (D) The afterdischarge (AD) threshold induced by first stimulus in rats treated with vehicle, menthol and PB (**P<0.01 compared with Ctrl, *n* = 10). (E) The seizure stage in fully kindled rats treated with vehicle, menthol and PB (**P<0.01 compared with Ctrl, *n* = 6). (F) Summary data showing afterdischarge duration in Ctrl, menthol and PB group (**P<0.01 compared with Ctrl, *n* = 6).

Network hyperactivity of the brain is the cause of epileptic seizures. Many antiepileptic agents exert anticonvulsant effect through inhibiting hyperactivity. We have used pentylenetetrazole (PTZ) model of epilepsy, a widely accepted method for evaluation of anticonvulsant drug, to examine whether menthol also inhibits *in vivo* hyperactivity. The target of PTZ is known to include GABA_A_Rs [26]. As shown in Fig. 5, clonic and tonic seizures were observed in mice within 30 min after PTZ injection. Administration of menthol (200 mg/kg, i.p.) significantly prolonged the latency to clonic (*p*<0.01 compared with Ctrl, Fig. 5A) and tonic seizures (*p*<0.05 compared with Ctrl, Fig. 5A). In addition, menthol markedly reduced the mortality of the mice (Fig. 5B). Therefore, menthol has an anticonvulsant effect in the PTZ mouse model, presumably through its potentiation on tonic GABAergic inhibition.

To further ascertain whether menthol inhibits hippocampal hyperactivity *in situ*, we investigated the effect of intracerebroventricular injection of menthol on hippocampal kindling, a more stable rat model of epilepsy. In these experiments, the anticonvulsive drug phenobarbital (PB) was used as a positive control. In normal rats prior to kindling, the afterdischarge threshold for rats injected with either menthol (780 µg in 5 µL) or PB (640 µg in 5 µL) was significantly higher than that observed in vehicle-injected rats (*p*<0.01 compared with Ctrl, Fig. 5D). For fully kindled rats (5 constitutive 5 class seizures by Racine's standard classification), we found that menthol or PB treatment significantly reduced the susceptibility of rats to seizure (*p*<0.01 compared with Ctrl, Fig. 5E) and the afterdischarge duration (Fig. 5C and 5F), as compared to those found in the vehicle-injected rats (Ctrl). Therefore, menthol exerts anticonvulsant effect in both PTZ and kindling models, consistent with the enhanced tonic GABAergic inhibition, which plays an important role in regulating network hyperactivity.

### The critical role of hydroxyl group in menthol enhancement of GABA_A_Rs

**Figure 6:**
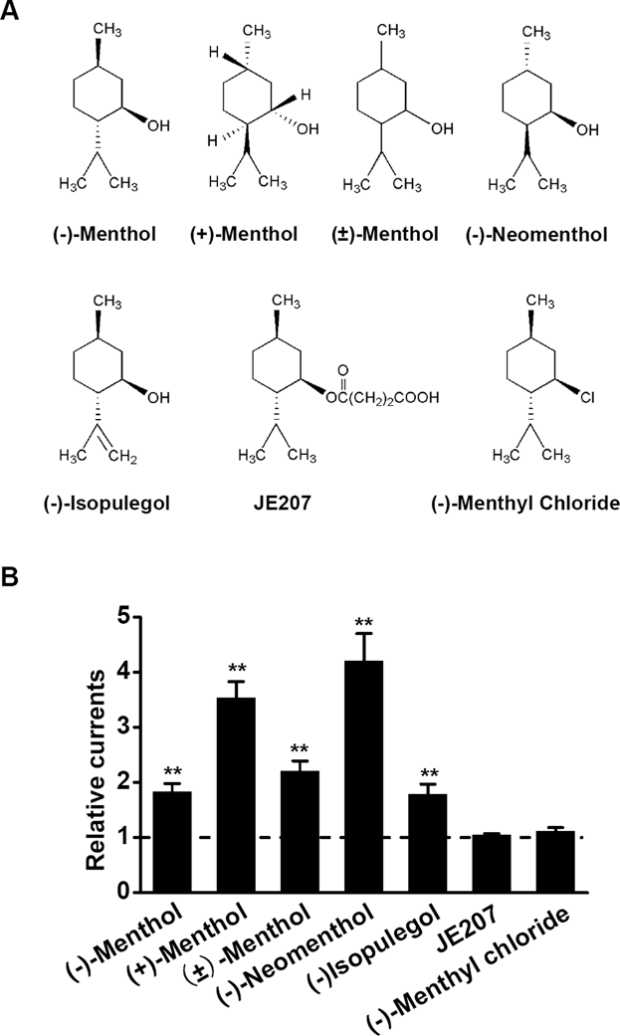
Effects of menthol isomers, (−)-isopulegol, JE207 and (−)-menthyl chloride on GABA_A_R. (A) Chemical structures of menthol isomers, (−)-isopulegol, JE207 and (−)-menthyl chloride. (B) Summary of data showing the relative currents induced by 1 µM GABA in the presence of 300 µM various menthol isomers, (−)-isopulegol, JE207 or (−)-menthyl chloride.

Finally, in order to further examine the structural basis of menthol modulation on GABA_A_Rs, we explored the structure-activity relationship of menthol enhancement on GABA_A_Rs. There are four main isomers of menthol: (−)-menthol, (+)-menthol, (±)-menthol and (−)-neomenthol (Fig. 6A). The menthol isomers all significantly enhanced *I*_GABA_ induced by 1 µM GABA in cultured hippocampal neurons (Fig. 6B). In addition, we tested another three structurally related chemicals of menthol, (−)-isopulegol, JE207 and (−)-menthyl chloride, on *I*_GABA_ induced by 1 µM GABA. As shown in Fig. 6B, (−)-isopulegol significantly enhanced *I*_GABA_, while JE207 and (−)-menthyl chloride, the hydroxyl substitutes of menthol, have no significant effect on *I*_GABA_. Therefore, these results indicate that the hydroxyl plays a critical role in menthol enhancement of GABA_A_Rs.

## Discussion

In cultured hippocampal neurons, we found that the enhancing effect of menthol occurred only when GABA concentration was ≤3 µM, suggesting that menthol preferentially acts on extrasynaptic high-affinity GABA_A_Rs. In accordance with the modulation of menthol on GABA responses in cultured neurons, we observed a differential effect of menthol on tonic GABA currents and GABAergic mIPSCs in CA1 pyramidal neurons of hippocampal slices. Notably, the tonic current activated by ambient low concentration of GABA was significantly enhanced by menthol, while the phasic GABAergic current mIPSC was not affected. Therefore, these results indicate that menthol selectively enhances tonic GABAergic inhibition in CA1 pyramidal neurons of the hippocampus.

The ambient GABA concentration in the extracellular space is estimated to be 0.8–2.9 µM [23], which is sufficient to activate a small percentage of high-affinity GABA_A_Rs. Our data show that menthol significantly suppressed spontaneous activity of cultured hippocampal neurons. Furthermore, hippocampal neurons in culture are capable of forming extensive synaptic networks that display physiological functions such as synaptic currents and zero Mg^2+^-induced epileptiform activity [21], [27]. Not surprisingly, the hyper-excitability of neurons perfused with Mg^2+^-free medium was also inhibited by the menthol treatment. In agreement with other studies on the activation of tonic GABAergic receptors by ambient GABA in cultured hippocampal neurons [6], [28], our results suggest that menthol suppresses the neuronal excitability mainly via enhancing GABA_A_R-mediated tonic inhibition in the hippocampus.

A recent study suggests that extrasynaptic GABA_A_Rs were optimally activated by ambient GABA under physiological conditions and a further increase in extracellular GABA concentration would not significantly enhance the effect of tonic inhibition on neuronal excitability [29]. Therefore, the functional potentiation of tonically activated GABA_A_Rs might be a promising therapeutic approach to treating diseases involving hyper-excitability such as epileptic seizures. Different animal models that reflect certain kinds of epilepsy are used to evaluate the effect of anticonvulsant drugs. Among which, PTZ has been reported to produce seizures by inhibiting GABAergic neurotransmission [30]. In the present study, therefore, PTZ seizure model was firstly used to examine the *in vivo* actions of menthol, which acts selectively at GABA_A_R-mediated tonic inhibition. Our results demonstrated that systematical administration of menthol exerts anticonvulsant effects by prolonging the latency of clonic and tonic seizures induced by PTZ. A previous study also showed that intraperitoneal administration of menthol caused ambulation-promoting effect in mice, suggesting that menthol could enter the brain and reach an effective concentration [17]. Given the capability of menthol to penetrate blood-brain barrier, as suggested by the previous [17] and the present data (Fig. 5A and 5B), it is likely that menthol intake may be potentially beneficial for lowering network hyperactivity under both normal and pathological conditions.

Kindling represents the propagation of the epileptic discharge to distal sites and the possible recruitment of those sites into the discharge, leading to enhanced sensitivity to focal electrical stimulation. Therefore, hippocampal kindling is a widely accepted model of temporal lobe epilepsy [31], [32], which has been validated as a reliable predictor of anticonvulsant drug efficacy [33]. We found that menthol increased the afterdischarge threshold, prolonged the afterdischarge duration, and reduced the seizure susceptibility of hippocampal kindled rats. These results, together with the anticonvulsant effect of menthol in PTZ-treated mice, strongly support a role of menthol-enhancing tonic inhibition in preventing epileptiform hyper-excitability.

There is mounting evidence for extrasynaptic GABA_A_Rs having subunit compositions different from those of synaptic receptors [3], [34]–[36]. The tonic GABAergic inhibition is mediated by α6δ-containing GABA_A_Rs in cerebellar granule cells [34], but by δ-subunit-containing and α5-subunit-containing GABA_A_Rs in dentate gyrus granule cells [34] and hippocampal pyramidal neurons [1], [35], respectively. Selective modulators of tonically activated receptor are valuable tools for investigating the function of tonic inhibition. Previous studies suggest that the α5 subunit is a specific subunit forming extrasynaptic receptors in hippocampal pyramidal neurons [35], [36]. In support of this assumption, selective decrease of tonic inhibition in both CA1 and CA3 pyramidal neurons of adult *gabra5* ^−/−^ mouse hippocampal slices was observed [37]. This selective decrease in tonic inhibition leads to epileptiform hyper-excitability in the CA3 pyramidal layer. Here, we demonstrate that menthol is a selective enhancer of tonic inhibition of hippocampal pyramidal neurons. Therefore, the suppression of neuronal hyper-excitability and epileptiform activity through selective enhancement of tonic inhibition in pyramidal neurons further confirm that tonic inhibition plays an important role in controlling the network excitability including both physiological oscillations and the pathological propagation of epileptiform activity. Perhaps it would be better to identify the subtype of activated GABA_A_Rs. However, this is not really relevant in this case as it is the concentration of ambient GABA that determines which receptors (e.g. tonic versus phasic) are activated by menthol and not menthol *per se*.

Finally, it would be of some interest to know something more about the mechanisms of menthol's effect on GABA_A_Rs. In this regard, a recent study examined menthol's actions on GABA_A_Rs compared to sedatives (benzodiazepines) and intravenous anesthetics (barbiturates, steroids, etomidate and propofol) [38]. The study indicates that menthol exerts its actions on GABA_A_Rs via sites distinct from benzodiazepines, steroids and barbiturates, and via sites important for modulation by propofol. This result is not unexpected given the apparent structural similarities between menthol and propofol (e.g. positioning of an isopropyl group adjacent to their respective hydroxyl groups). Interestingly, propofol at clinically-relevant concentrations selectively enhances tonic currents activated by GABA at low concentrations in hippocampal neurons [6], providing additional evidence favoring the idea that menthol and propofol may modulate GABA_A_Rs with a similar molecular mechanism.

In conclusion, the present results suggest that menthol selectively enhances tonic inhibition mediated by high-affinity, slowly desensitizing GABAARs in CA1 pyramidal neurons of rat hippocampus, leading to inhibition of *in vitro* neuronal excitability and *in vivo* network hyper-excitability of the hippocampus. Our results, therefore, reveal a novel role of menthol in the mammalian CNS and underscore the importance of tonic inhibition in controlling neuronal excitability.

## Materials and Methods

### Cell culture and electrophysiological recording

Animals were treated in accordance with the Animal Care and Use Committee of the Institute of Neuroscience. Primary hippocampal neurons were cultured as previously described [39]. Whole-cell or cell-attached recordings were made using a patch-clamp amplifier (Axon 200B, Axon Instruments, Foster City, CA, USA). The standard extracellular solution contained (mM): 150 NaCl, 5 KCl, 1 MgCl_2_, 2 CaCl_2_, 10 N-hydroxyethylpiperazine-NV-2-ethanesulphonic acid (HEPES), and 10 glucose (pH 7.3 with Tris-base, 325–330 milliosmolar with sucrose). The pipette solution with the high Cl^−^ concentration was composed of (mM): 120 KCl, 30 NaCl, 1 MgCl_2_, 0.5 CaCl_2_, 5 EGTA, 2 Mg-ATP, 10 HEPES, pH 7.3 adjusted with Tris-base. When *I-V* relationships of the currents were examined, tetrodotoxin (TTX, 300 nM) and CdCl_2_ (100 µM) were added to the standard extracellular solution and K^+^ was replaced with Cs^+^ in the pipette solution. The pipette solution with the low Cl^−^ concentration contained (mM): 120 CsOH, 30 NaCl, 0.2 EGTA, 2 Mg-ATP, 10 HEPES, pH was adjusted to 7.3 with gluconic acid. Additionally, action-potential discharges were recorded using whole-cell current-clamp method with the K-gluconate pipette solution containing (mM) 150 K-gluconate, 9 NaCl, 1 MgCl_2_, 0.2 EGTA, 10 HEPES, pH 7.3 adjusted with Tris-base.

Membrane currents were sampled and analyzed using a Digidata 1320A interface and a personal computer with Clampex and Clampfit software (Version 9.0.1, Axon Instruments). Unless otherwise noted, the membrane potential was held at −50 mV for all whole-cell current recordings, and the patch potential was held at 0 mV for recording firing activity under cell-attached voltage-clamp mode. In cell-attached voltage-clamp recording, firing rate was evaluated from the mean interspike interval, and analyzed with the MiniAnalysis 6.0.1 program (Synaptosoft, Decatur, GA). Concentration-response curves were drawn according to a modified Michaelis-Menten equation by the method of least-squares (the Newton-Raphson method) after normalizing the amplitude of the response: *I* = *I*_max_ *C^h^*/(*C^h^*+*EC*_50_ *^h^*), where *I* is the normalized value of the current, *I*_max_ is the maximal response, *C* is the drug concentration, EC_50_ is the concentration which induces the half-maximal response and *h* is the apparent Hill coefficient.

### Brain slices preparation and electrophysiological recordings

Sprague-Dawley rats (14–21 days after birth) were anesthetized with halothane. Brains were quickly removed and 400 µm coronal hippocampal slices were cut in well-oxygenated ice-cold artificial cerebrospinal fluid (ACSF) containing 126 mM NaCl, 2.5 mM KCl, 10 mM d-glucose, 2 mM MgSO_4_, 2 mM CaCl_2_, 1.25 mM NaH_2_PO_4_, and 26 mM NaHCO_3_ (pH 7.3–7.4 when bubbled with 95% O_2_ and 5% CO_2_). Immediately after cutting, slices were incubated at 36±1°C for 1 h, followed by room temperature (22–25°C) incubation in oxygenated ACSF before recording. Whole-cell patch clamp recordings were made from hippocampal CA1 pyramidal neurons under control by infrared-differential interference contrast (IR-DIC) video microscope (Olympus, BX51WI). The holding potential was −60 mV. Patch pipettes had open tip resistances of 3–5 MΩ when filled with an intracellular solution that contained 140 mM CsCl, 10 mM HEPES, 1 mM MgCl_2_, 0.1 mM EGTA, 4 mM NaCl, 2 mM Mg-ATP, 0.3 mM Na-GTP, 5 mM lidocaine N-ethylbromide (QX-314) (pH 7.3, 280–290 milliosmolar). The extracellular recording solution contained 3 mM kynurenic acid and 1 µM TTX were used to block ionotropic glutamate receptors and action potential, respectively. During the experiments, 5 µM GABA was added to enlarge the basal tonic currents[40]. To evaluate GABA tonic currents, 30 µM BMI was used. The MiniAnalysis 6.0.1 program (Synaptosoft, Decatur, GA) was used to analyze mIPSCs.

### PTZ seizures test

Male ICR mice (20–25 g) received daily administration of 200 mg/kg menthol (1 ml/kg i.p.) for drug group and saline for control group for 3 days. Menthol was suspended in 1% Tween 80/distilled water for i.p. injection. Thirty minutes after the last injection, PTZ was administered at 85 mg/kg, i.p [41]. This dose produces the following behavioral changes: myoclonus, defined as a whole-body twitch; clonic seizures, manifested by clonic spasms often followed by stupor or unusual posturing; and tonic seizures consisted of tonic hind limb extension, which is usually the lethal component in approximately 50% of the mice under normal conditions. The latencies to the first clonic seizure and to the tonic extension as well as mortality were visually evaluated during 30 min after PTZ administration.

### Kindling procedure and anticonvulsant test

Adult male Sprague-Dawley rats weighing 200–250 g were maintained on a 12 h light/dark cycle with ad libitum access to food and water. Under chloral hydrate (250 mg/kg; i.p.) anesthesia, bipolar electrode of stainless steel used for stimulation and recording was stereotaxically implanted in the right hippocampal CA1 (4.0 mm posterior to bregma, 2.6 mm lateral to the midline, 2.5 mm below dura). Four screws were inserted into the skull through a drilled hole without piercing the dura. One served as the reference (6.0 mm posterior to bregma, 3.0 mm left lateral to the midline) in the electroencephalogram (EEG) recording. Cannula was implanted into the left lateral ventricle (0.8 mm posterior to bregma, 1.5 mm lateral to the midline, and 4.0 mm below the skull surface) for drug infusion. Cannula, electrodes and screws were fixed with a mixture of acrylic and dental cement. After a postoperative recovery period of at least 7 days, the electroencephalographic seizure threshold was determined by application of a 1 s train of 1 ms monophasic rectangular pulses at 60 Hz beginning at 50 µA. The 25 µA steps were administered at 2 min interval until an afterdischarge lasting at least 5 s was detected. Drugs were administrated by intracerebroventricular injection at 20 min before afterdischarge threshold test. The intensity of afterdischarge threshold plus 100 µA was administered twice a day during following days. The behavioral progression of kindling-induced seizures was scored according to Racine's standard classification [32]: 0, no reaction; 1, stereotype mounting, eye blinking and/or mild facial clonus; 2, head nodding and/or several facial clonus; 3, myoclonic jerks in the forelimbs; 4, clonic convulsions in the forelimbs with rearing; and 5, generalized clonic convulsions associated with loss of balance. Fully kindled was defined by the seizure occurrence of three consecutive class 5.

For anticonvulsant test, rats were received twice a day stimulations without drug administration until the animals reached 5 constitutive 5 class seizures. Rats were acutely administered with vehicle (5 µl DMSO), menthol (780 µg in 5 µl DMSO solution) and PB (640 µg in 5 µl DMSO solution), respectively, by intracerebroventricular injection 20 min before stimulation to test the class of seizures.

### Drugs

All drugs were purchased from Sigma except that the compound JE207 was kindly provided by Kunming Institute of Botany, Chinese Academy of Sciences. For the electrophysiological experiments, the tested drugs were initially dissolved as concentrated stock solutions in DMSO and subsequently diluted to the desired concentration in perfusion solution.

### Statistical analysis

Group data are presented as mean±s.e.m. Statistical comparisons were made with Student's t-test or One-way ANOVA. P<0.05 was considered statistically significant.

## Supporting Information

Figure S1 Lack of effect of menthol on action-potential discharge. (A) Representative traces showing the sustained action-potential discharges evoked by injection of various depolarizing current (30 pA, 110 pA and 190 pA) in cultured hippocampal neurons in the absence or presence of 300 µM menthol. Synaptic transmission was blocked by CNQX (10 µM), D-AP5 (20 µM), BMI (10 µM) and STN (1 µM). (B) The frequency of action-potential discharge evoked by various current intensity (30–210 pA, 500 ms) in the absence or presence of 300 µM menthol. n = 11–15.
(1.61 MB TIF)

Figure S2 Lack of TRPM8 expression in cultured hippocampal neurons and hippocampus tissue. (A) Reverse transcriptase-PCR primers to probe TRPM8. (B) Agarose gel electrophoresis of mRNA products obtained after amplification of base pair sequence specific for TRPM8 with reverse transcriptase-PCR. Evaluation of the constitutively expressed actin gene was included as a quality control for the cDNA.
(2.27 MB TIF)

Figure S3 Lack of menthol effect on currents mediated by ionotropic glutamate receptors. (A) Representative traces showing the currents evoked by 100 µM glutamate in the absence or presence of 300 µM menthol. All experiments were performed in Mg^2+^-free extracellular solution containing 1 µM glycine. (B) Summary results from all experiments similar to that shown in A, illustrating the lack of effect of menthol on currents mediated by ionotropic glutamate receptors. n = 5. (C) Representative traces showing mEPSCs in the absence or presence of 300 µM menthol. Averaged mEPSCs in the absence or presence of 300 µM menthol are shown in the pane. Miniature EPSCs were recorded in the presence of 300 nM TTX plus 10 µM BMI and were completely blocked by 3 µM D-AP5 and 10 µM CNQX. (D) Normalized cumulative curves showing the effect of menthol on the amplitude of mEPSCs from the sample neuron. (E) Normalized cumulative curves showing the effect of menthol on the frequency of mEPSCs from the sample neuron. (F) Summary data showing normalized amplitude, frequency of mEPSCs in the presence of menthol (n = 5). Dashed line indicates the control values without menthol treatment.
(9.40 MB TIF)

## References

[1] Farrant M, Nusser Z (2005). Variations on an inhibitory theme: phasic and tonic activation of GABA_A_ receptors.. Nat Rev Neurosci.

[2] Semyanov A, Walker MC, Kullmann DM (2003). GABA uptake regulates cortical excitability via cell type-specific tonic inhibition.. Nat Neurosci.

[3] Nusser Z, Mody I (2002). Selective modulation of tonic and phasic inhibitions in dentate gyrus granule cells.. J Neurophysiol.

[4] Brickley SG, Cull-Candy SG, Farrant M (1996). Development of a tonic form of synaptic inhibition in rat cerebellar granule cells resulting from persistent activation of GABA_A_ receptors.. J Physiol.

[5] Liu QY, Vautrin J, Tang KM, Barker JL (1995). Exogenous GABA persistently opens Cl^−^ channels in cultured embryonic rat thalamic neurons.. J Membr Biol.

[6] Bai D, Zhu G, Pennefather P, Jackson MF, MacDonald JF (2001). Distinct functional and pharmacological properties of tonic and quantal inhibitory postsynaptic currents mediated by gamma-aminobutyric acid(A) receptors in hippocampal neurons.. Mol Pharmacol.

[7] Chadderton P, Margrie TW, Hausser M (2004). Integration of quanta in cerebellar granule cells during sensory processing.. Nature.

[8] Semyanov A, Walker MC, Kullmann DM, Silver RA (2004). Tonically active GABA_A_ receptors: modulating gain and maintaining the tone.. Trends Neurosci.

[9] Houser CR, Esclapez M (2003). Downregulation of the alpha5 subunit of the GABA_A_ receptor in the pilocarpine model of temporal lobe epilepsy.. Hippocampus.

[10] Croteau RB, Davis EM, Ringer KL, Wildung MR (2005). (−)-Menthol biosynthesis and molecular genetics.. Naturwissenschaften.

[11] Peier AM, Moqrich A, Hergarden AC, Reeve AJ, Andersson DA (2002). A TRP channel that senses cold stimuli and menthol.. Cell.

[12] Zuker CS (2002). Neurobiology: a cool ion channel.. Nature.

[13] Tsuzuki K, Xing H, Ling J, Gu JG (2004). Menthol-induced Ca^2+^ release from presynaptic Ca^2+^ stores potentiates sensory synaptic transmission.. J Neurosci.

[14] Thebault S, Lemonnier L, Bidaux G, Flourakis M, Bavencoffe A (2005). Novel role of cold/menthol-sensitive transient receptor potential melastatine family member 8 (TRPM8) in the activation of store-operated channels in LNCaP human prostate cancer epithelial cells.. J Biol Chem.

[15] Wasner G, Schattschneider J, Binder A, Baron R (2004). Topical menthol–a human model for cold pain by activation and sensitization of C nociceptors.. Brain.

[16] Namer B, Seifert F, Handwerker HO, Maihofner C (2005). TRPA1 and TRPM8 activation in humans: effects of cinnamaldehyde and menthol.. Neuroreport.

[17] Umezu T, Sakata A, Ito H (2001). Ambulation-promoting effect of peppermint oil and identification of its active constituents.. Pharmacol Biochem Behav.

[18] Sipila ST, Huttu K, Soltesz I, Voipio J, Kaila K (2005). Depolarizing GABA acts on intrinsically bursting pyramidal neurons to drive giant depolarizing potentials in the immature hippocampus.. J Neurosci.

[19] Perkins KL (2006). Cell-attached voltage-clamp and current-clamp recording and stimulation techniques in brain slices.. J Neurosci Methods.

[20] Walther H, Lambert JD, Jones RS, Heinemann U, Hamon B (1986). Epileptiform activity in combined slices of the hippocampus, subiculum and entorhinal cortex during perfusion with low magnesium medium.. Neurosci Lett.

[21] Sombati S, Delorenzo RJ (1995). Recurrent spontaneous seizure activity in hippocampal neuronal networks in culture.. J Neurophysiol.

[22] Hall AC, Turcotte CM, Betts BA, Yeung WY, Agyeman AS (2004). Modulation of human GABA_A_ and glycine receptor currents by menthol and related monoterpenoids.. Eur J Pharmacol.

[23] Lerma J, Herranz AS, Herreras O, Abraira V, Martin del Rio R (1986). In vivo determination of extracellular concentration of amino acids in the rat hippocampus. A method based on brain dialysis and computerized analysis.. Brain Res.

[24] Mtchedlishvili Z, Kapur J (2006). High-affinity, slowly desensitizing GABA_A_ receptors mediate tonic inhibition in hippocampal dentate granule cells.. Mol Pharmacol.

[25] Mody I, De Koninck Y, Otis TS, Soltesz I (1994). Bridging the cleft at GABA synapses in the brain.. Trends Neurosci.

[26] Huang RQ, Bell-Horner CL, Dibas MI, Covey DF, Drewe JA (2001). Pentylenetetrazole-induced inhibition of recombinant gamma-aminobutyric acid type A (GABA_A_) receptors: mechanism and site of action.. J Pharmacol Exp Ther.

[27] Mangan PS, Kapur J (2004). Factors underlying bursting behavior in a network of cultured hippocampal neurons exposed to zero magnesium.. J Neurophysiol.

[28] Yeung JY, Canning KJ, Zhu G, Pennefather P, MacDonald JF (2003). Tonically activated GABA_A_ receptors in hippocampal neurons are high-affinity, low-conductance sensors for extracellular GABA.. Mol Pharmacol.

[29] Yeh JH, Jeng CJ, Chen YW, Lin HM, Wu YS (2005). Selective enhancement of tonic inhibition by increasing ambient GABA is insufficient to suppress excitotoxicity in hippocampal neurons.. Biochem Biophys Res Commun.

[30] Okada R, Negishi N, Nagaya H (1989). The role of the nigrotegmental GABAergic pathway in the propagation of pentylenetetrazol-induced seizures.. Brain Res.

[31] Goddard GV, McIntyre DC, Leech CK (1969). A permanent change in brain function resulting from daily electrical stimulation.. Exp Neurol.

[32] Racine RJ (1972). Modification of seizure activity by electrical stimulation. II. Motor seizure.. Electroencephalogr Clin Neurophysiol.

[33] Majkowski J (1999). Kindling: clinical relevance for epileptogenicity in humans.. Adv Neurol.

[34] Stell BM, Brickley SG, Tang CY, Farrant M, Mody I (2003). Neuroactive steroids reduce neuronal excitability by selectively enhancing tonic inhibition mediated by delta subunit-containing GABA_A_ receptors.. Proc Natl Acad Sci U S A.

[35] Caraiscos VB, Elliott EM, You-Ten KE, Cheng VY, Belelli D (2004). Tonic inhibition in mouse hippocampal CA1 pyramidal neurons is mediated by alpha5 subunit-containing gamma-aminobutyric acid type A receptors.. Proc Natl Acad Sci U S A.

[36] Tsunashima K, Schwarzer C, Kirchmair E, Sieghart W, Sperk G (1997). GABA_A_ receptor subunits in the rat hippocampus III: altered messenger RNA expression in kainic acid-induced epilepsy.. Neuroscience.

[37] Glykys J, Mody I (2006). Hippocampal network hyperactivity after selective reduction of tonic inhibition in GABA_A_ receptor alpha5 subunit-deficient mice.. J Neurophysiol.

[38] Watt EE, Betts BA, Kotey FO, Humbert DJ, Griffith TN (2008). Menthol shares general anesthetic activity and sites of action on the GABA_A_ receptor with the intravenous agent, propofol.. Eur J Pharmacol.

[39] Gao J, Duan B, Wang DG, Deng XH, Zhang GY (2005). Coupling between NMDA receptor and acid-sensing ion channel contributes to ischemic neuronal death.. Neuron.

[40] Wei W, Faria LC, Mody I (2004). Low ethanol concentrations selectively augment the tonic inhibition mediated by delta subunit-containing GABA_A_ receptors in hippocampal neurons.. J Neurosci.

[41] White HSWJ, Franklin MR, Swinyard EA, Wolf HA, Levy RH, Mattson RH, Meldrum BS (1995). Antiepileptic drugs, Ed 4.

