# Supplementary figures and images for: A-Type GABA Receptor as a Central Target of TRPM8 Agonist Menthol
Source: PLoS One. 2008 Oct 13;3(10):e3386. doi: 10.1371/journal.pone.0003386 (PMC2560999; doi:10.1371/journal.pone.0003386)

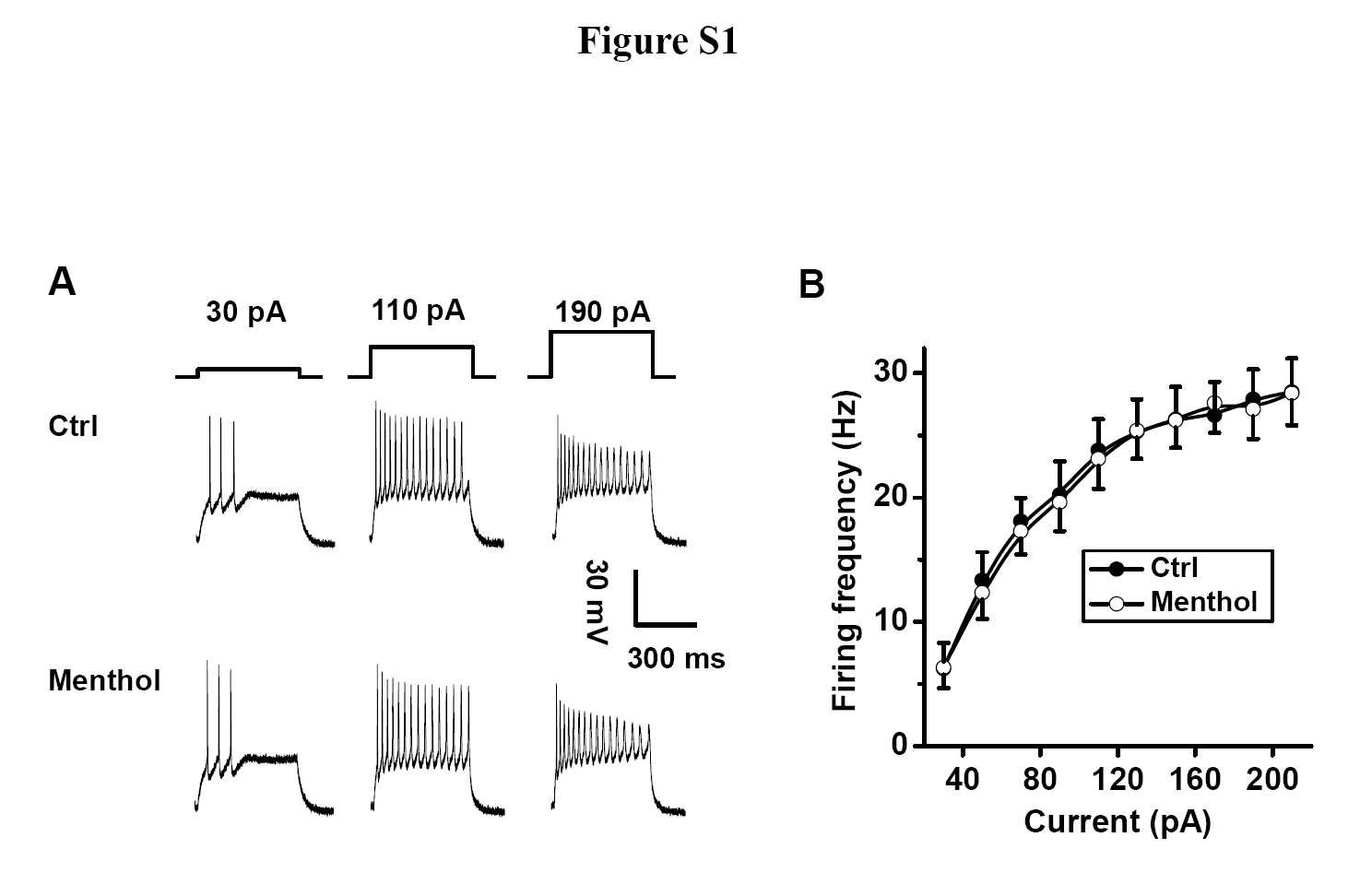

Supplement: Figure S1 — Lack of effect of menthol on action-potential discharge. (A) Representative traces showing the sustained action-potential discharges evoked by injection of various depolarizing current (30 pA, 110 pA and 190 pA) in cultured hippocampal neurons in the absence or presence of 300 µM menthol. Synaptic transmission was blocked by CNQX (10 µM), D-AP5 (20 µM), BMI (10 µM) and STN (1 µM). (B) The frequency of action-potential discharge evoked by various current intensity (30–210 pA, 500 ms) in the absence or presence of 300 µM menthol. n = 11–15. (1.61 MB TIF) [file pone.0003386.s001.tif]

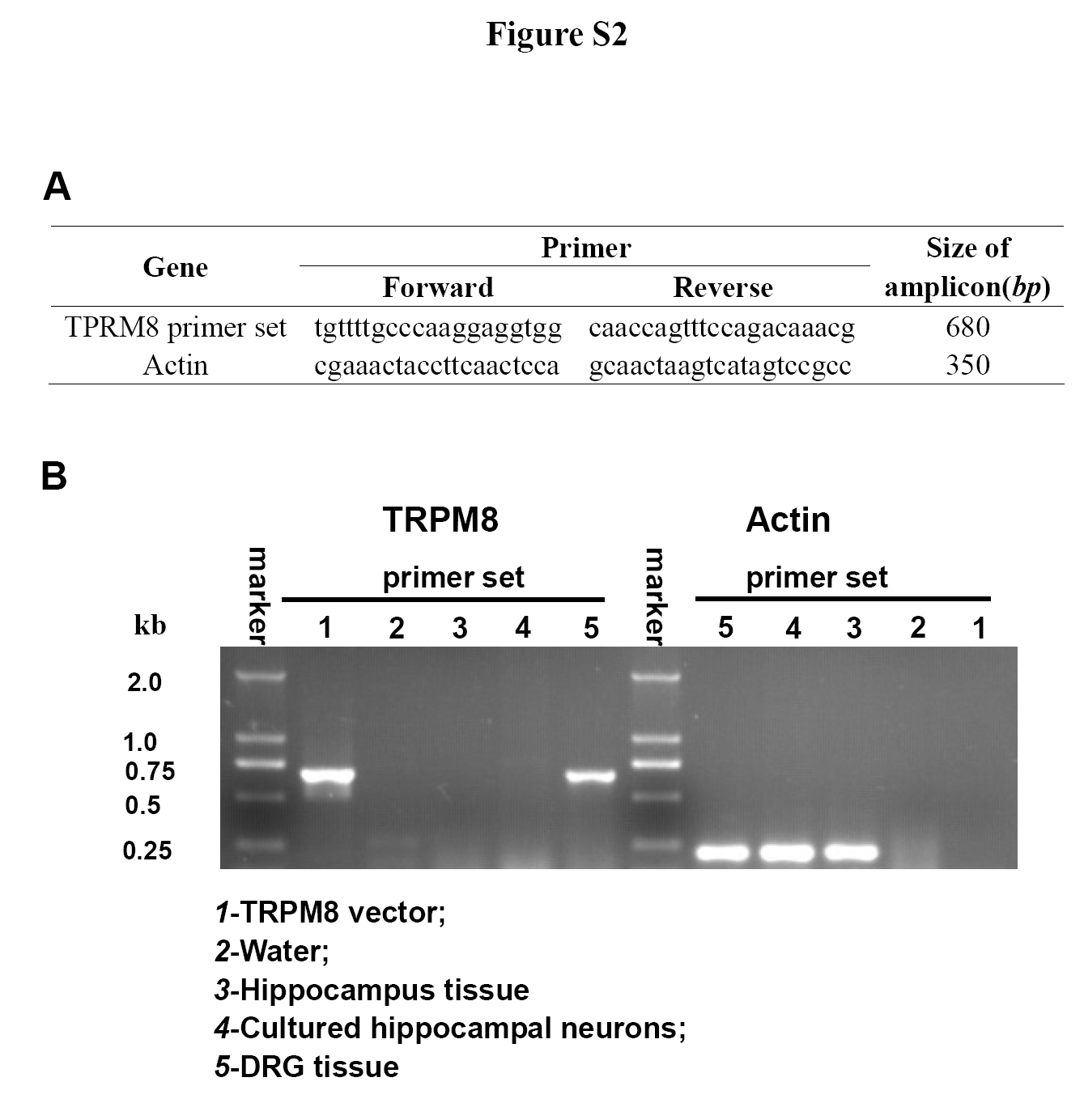

Supplement: Figure S2 — Lack of TRPM8 expression in cultured hippocampal neurons and hippocampus tissue. (A) Reverse transcriptase-PCR primers to probe TRPM8. (B) Agarose gel electrophoresis of mRNA products obtained after amplification of base pair sequence specific for TRPM8 with reverse transcriptase-PCR. Evaluation of the constitutively expressed actin gene was included as a quality control for the cDNA. (2.27 MB TIF) [file pone.0003386.s002.tif]

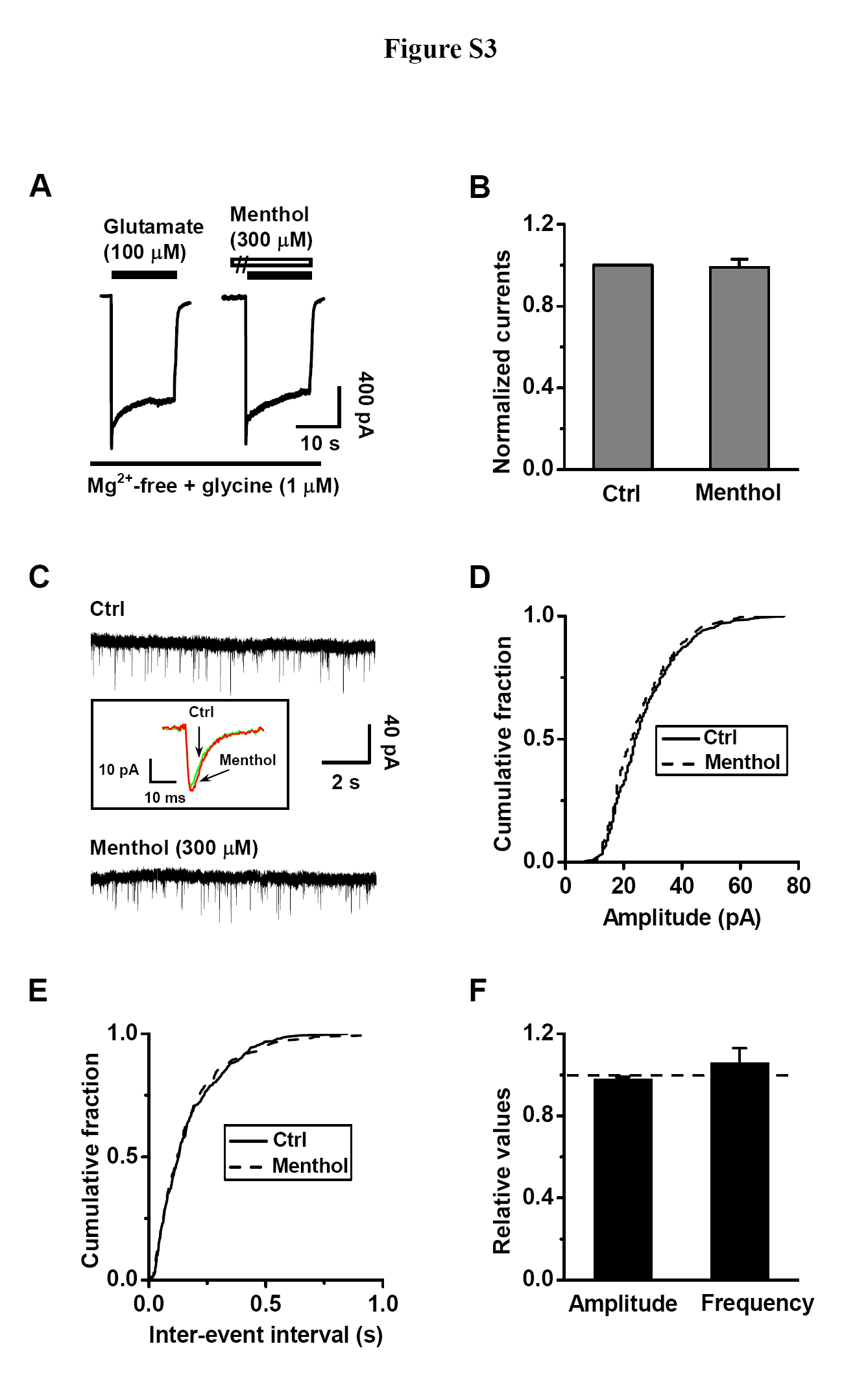

Supplement: Figure S3 — Lack of menthol effect on currents mediated by ionotropic glutamate receptors. (A) Representative traces showing the currents evoked by 100 µM glutamate in the absence or presence of 300 µM menthol. All experiments were performed in Mg2+-free extracellular solution containing 1 µM glycine. (B) Summary results from all experiments similar to that shown in A, illustrating the lack of effect of menthol on currents mediated by ionotropic glutamate receptors. n = 5. (C) Representative traces showing mEPSCs in the absence or presence of 300 µM menthol. Averaged mEPSCs in the absence or presence of 300 µM menthol are shown in the pane. Miniature EPSCs were recorded in the presence of 300 nM TTX plus 10 µM BMI and were completely blocked by 3 µM D-AP5 and 10 µM CNQX. (D) Normalized cumulative curves showing the effect of menthol on the amplitude of mEPSCs from the sample neuron. (E) Normalized cumulative curves showing the effect of menthol on the frequency of mEPSCs from the sample neuron. (F) Summary data showing normalized amplitude, frequency of mEPSCs in the presence of menthol (n = 5). Dashed line indicates the control values without menthol treatment. (9.40 MB TIF) [file pone.0003386.s003.tif]
